# Personality and social support as determinants of entrepreneurial intention. Gender differences in Italy

**DOI:** 10.1371/journal.pone.0199924

**Published:** 2018-06-28

**Authors:** Monica Molino, Valentina Dolce, Claudio Giovanni Cortese, Chiara Ghislieri

**Affiliations:** Department of Psychology, University of Turin, Turin, Italy; Leeds University Business School, UNITED KINGDOM

## Abstract

The interest in the promotion of entrepreneurship is significantly increasing, particularly in those countries, such as Italy, that suffered during the recent great economic recession and subsequently needed to revitalize their economy. Entrepreneurial intention (EI) is a crucial stage in the entrepreneurial process and represents the basis for consequential entrepreneurial actions. Several research projects have sought to understand the antecedents of EI. This study, using a situational approach, has investigated the personal and contextual determinants of EI, exploring gender differences. In particular, the mediational role of general self-efficacy between internal locus of control (LoC), self-regulation, and support from family and friends, on the one hand, and EI, on the other hand, has been investigated. The study involved a sample of 658 Italian participants, of which 319 were male and 339 were female. Data were collected with a self-report on-line questionnaire and analysed with SPSS 23 and Mplus 7 to test a multi-group structural equation model. The results showed that self-efficacy totally mediated the relationship between internal LoC, self-regulation and EI. Moreover, it partially mediated the relationship between support from family and friends and EI. All the relations were significant for both men and women; however, our findings highlighted a stronger relationship between self-efficacy and EI for men, and between support from family and friends and both self-efficacy and EI for women. Findings highlighted the role of contextual characteristics in addition to personal ones in influencing EI and confirmed the key mediational function of self-efficacy. As for gender, results suggested that differences between men and women in relation to the entrepreneur role still exist. Practical implications for trainers and educators are discussed.

## Introduction

In the last few years, the interest in the promotion of entrepreneurship significantly has increased in many advanced economies. Start-ups and new businesses seem to be essential in order to ameliorate economic conditions, to create new job positions, and to give value to societies. Without a doubt, a new business provides the market economy with innovation and vitality [1]. Thus, entrepreneurship has received an increasing interest in different fields of research and practice. From a practice point of view, consultants suggest fostering business creation through a supportive ecosystem characterized by the presence of incubators, accelerators, technological parks, co-working spaces, private and public investors (business angels, venture capitalist, etc.) and specific services aimed at supporting new entrepreneurs [2–4].

Although with a certain delay in comparison with other advanced economies, in the midst of these international considerations, in 2012, the Italian Government introduced the “2.0 decree” in order to promote business creation, proposing many measures to sustain start-ups [2].

In the literature, various definitions have been adopted for the purposes of discussing entrepreneurship; for instance, Shane [5] referred to it as “an activity that involves the discovery, evaluation and exploitation of opportunities to introduce new goods and services, ways of organizing, markets, processes, and raw materials through organizing efforts that previously had not existed” ([5], p. 4). The psychological research seeks to explain the phenomenon by mainly considering entrepreneurship as a human fact [6]. Initially, psychologists treated the topic by focusing principally on personality characteristics and motivations, while observing entrepreneurship as a state of being, rather than a process of becoming [7]. Recently, from a multidisciplinary perspective, many scholars have dealt with the topic by paying attention not only to the personal and motivational characteristics but also to social, cultural, organizational and economic factors [6, 8]. This study considers entrepreneurial intention (EI) as an opening phase in the entrepreneurial process, in order to investigate its determinants by considering two personal factors, internal locus of control (LoC) and self-regulation, and one contextual factor, perceived support from family and friends, with the mediation of general self-efficacy, among men and women.

### Entrepreneurial intention

EI represents the first fundamental step in creating a business; entrepreneurship indeed could be described as a process defined in four stages [1, 9]. First of all, it is fundamental that EI is sustained by almost one business idea; secondly, it must involve entrepreneurial choice; thirdly, it requires a planning project phase; fourthly, a new business should be created, which is followed by entrepreneurial success and finally by the development of an enterprise [1]. The process can be represented as a bottleneck: only some of the business ideas become business projects; furthermore, only certain business projects, i.e., those that have been exposed to start-up tests, move into action; finally, only a few of these projects succeed to effectively become enterprises [9, 10].

The EI phase is one of the most significant areas of interest concerning the entrepreneurial theme [6, 11, 12], which is essential for every aspiring start-upper, since without it any future enterprise does not exist. This phase is mainly played out in the aspiring start-upper’s mind; only later on does this intention turn into entrepreneurial choice [6]. In general terms, “intentionality is a state of mind directing a person’s attention (and therefore experience and action) toward a specific object (goal) or a path in order to achieve something (means)” ([13], p. 442).

Among scholars, interest in EI has rapidly growth [14, 15]. Liñán and Fayolle [15] proposed an interesting review of the literature on entrepreneurial intentions identifying five main research areas: 1) the core EI model; 2) the role of personal-level variables in the configuration of EI; 3) the relationship between entrepreneurship education and intentions; 4) the role of context and institutions; and 5) the link between intention and behaviour in the entrepreneurial process.

In the last 20 years, many models and theories have been developed to explain EI [6, 16]: the Theory of Planned Behaviour (TPB) [17, 18]; the Implementing Entrepreneurial Ideas (IEI) model [13]; the Shapero's Entrepreneurial Event (SEE) model [19]; and, more recently, the Lüthje and Franke’s model (LFM) [20].

According to the TPB [18], intention is a function of subjective norm, perceived behavioural control and attitude toward the behaviour, with behaviour as the result of intention. The Bird’s IEI model [13] was developed taking into account the Fishbein and Ajzein’s Theory [21], according to which intentions are conceptualized as functions of beliefs, which provide a link between beliefs and subsequent behaviours. Bird [13] claimed that the intentional entrepreneurial process begins in response to a combination of both personal (prior experience, personality, abilities) and contextual factors (social, political and economic variables). “Personal and social contexts interact with rational and intuitive thinking during the formulation of entrepreneurial intentions” ([13], p. 443). Rational/analytic thinking (goal-directed behaviour) underlies the creation of a business plan, opportunity analysis and goal-setting, while intuitive/holistic thinking (vision) furthers the entrepreneur’s perseverance and, in general terms, structures the action and intention [13].

According to the Shapero’s SEE model [19], three factors are crucial for EI: perceived desirability, perceived feasibility and propensity to act: perceived desirability can be defined as a strong attractiveness towards a business venture, while perceived feasibility indicates the extent to which people are confident about creating a business, and, finally, propensity to act concerns the disposition to act by taking into account opportunities [16].

More recently, Lüthje and Franke [20] developed a model that combines personality traits and contextual factors (support and barriers); in contrast to the SEE model and the TPB, the LFM considers exogenous factors able to directly affect EI [20].

Considering the Liñán and Fayolle’s classification [15], our study sought to contribute to the second and fourth categories, investigating the role of personal- and context-level variables as determinants of EI. The focus was, particularly, on self-regulation as personality trait and support from family and friends as contextual factor, two variables few investigated to date. Among the models presented in literature, for our purpose we considered the Boyd and Vozikis’ model [7], itself an evolution of the Bird’s model [13], which regards self-efficacy as a key mediator between contextual and personal characteristics and EI, and the previously mentioned LFM [20], which supposed a direct link between contextual factors and EI. As a more recent approach, the LFM has been applied in few studies so far (e.g., [16, 20, 22]), although the model provides a broad framework for the investigation of the antecedents of EI [16].

### Determinants of entrepreneurial intention

In the literature, attention has been given to the antecedents of EI by considering different personal and contextual factors that could impact it. Regarding personal factors, taking into account psychological aspects in order to explore entrepreneurship from a person-oriented perspective is diffusely sustained [23]. Several studies have discussed the effects of personality traits on EI [16, 24], such as LoC [20, 25], risk propensity [20, 24, 26], conscientiousness, openness to experience, emotional stability, extraversion [24] and self-efficacy [27, 28]. Furthermore, entrepreneurial passion [29, 30], creativity [31], emotional intelligence and proactive personality [26] are other factors that have been considered by scholars.

In this study, internal LoC and self-regulation are the two personal characteristics examined. LoC refers to how individuals attribute their achievements and their failures, differing between external and internal reinforcements [32], which lead to two types of LoC. Internal LoC is typical of those who explain events and facts that happen to them through self-behaviour, choices and responsibility. External LoC is typical of those who attribute the cause of an event to luck, fate or powerful, actors beyond an individual’s control [32]. Brockhaus, in 1975 [33], found that perceived LoC could be a predictor of EI, while Lüthje and Franke [20] concluded that personality traits, such as LoC and risk propensity, affect the attitude towards entrepreneurship. Nevertheless, in recent studies and meta-analyses about personality predictors of EI [28, 34], the role of LoC has been little highlighted.

Self-regulation is the second personal characteristic considered in this study. It refers to the sense of control over one’s own thoughts, motivations and behaviours [35], which involves being able to set goals, monitor results in relation to a personal internal standard, evaluate possible discrepancies and organize corrective solutions. In a multi-stage entrepreneurial process, the combination of “promotion-driven and prevention-driven motives, beliefs and behaviours [is] needed for entrepreneurial success” ([10], p. 208). Specifically, creativity in generating alternative business ideas, driven by dreams and aspirations, openness to change and felt presence of positive outcomes, may coexist with responsibility, sense of duty and strategic vigilance in order to avoid mistakes (fundamental during the phase when the business idea is screened) [10, 36, 37].

Brockner and colleagues [10] distinguished self-regulation according to promotion or prevention focus, arguing that, for entrepreneurial success, both foci are needed. From the start of the entrepreneurial process, entrepreneurs seek to bring themselves into alignment with their dreams and aspirations (promotion focus), but also with their sense of duty and responsibility (prevention focus) [10]. Indeed, the entrepreneurial process is characterized by stages (e.g., the generating ideas phase), in which the perception of positive outcomes is salient (promotion focus), and others (e.g., screening ideas), in which the perception of potential losses is more relevant [10].

Unlike self-efficacy, self-regulation has only recently been studied in relation to the entrepreneurial process [10, 29, 38]. Molino and colleagues [29], in a recent study, compared entrepreneurs with employees and students, finding that entrepreneurs have a significantly higher level of self-regulation than the other two groups. Pihie and Bagheri [38], in their study on university students, developed a model in which self-regulation mediated the relationship between self-efficacy and EI. In this study, we hypothesized that internal LoC and self-regulation have a positive relation with EI.

Hypothesis 1: Internal LoC and self-regulation have a positive relation with EI.

According to Boyd and Vozikis’ model [7], the influence of personal and contextual characteristics on EI could be mediated by self-efficacy, which has been defined as “people’s beliefs about their capabilities to produce designated levels of performance that exercise influence over events that affect their lives” ([39], p. 71). Boyd and Vozikis highlighted the key role of self-efficacy, since beliefs about one’s own possibilities to succeed when launching a business can influence EI development [7], while mediating the relationship of both personal and contextual factors with EI. Moreover, self-efficacy is able to buffer the relation between EI and entrepreneurial actions [7].

In this study, we considered the direct link between general self-efficacy and EI and its mediational role between personality factors (self-regulation and internal LoC) and context, on the one hand, and EI, on the other hand. Self-efficacy is indeed a motivational construct [28], while, in line with scholars’ arguments [40], motivation is an important mediator between individual traits and entrepreneurial outcomes.

A debate about the potential overlap between self-efficacy and other related constructs, such as LoC, is present in literature: for example, Judge and colleagues [41] found that a single latent factor may explain the relationships between measures of self-efficacy, self-esteem, neuroticism and LoC. At the same time, other authors have argued that it would be theoretically reasonable to expect an effect of LoC on self-efficacy [42], since perceived control on the environment has been found to be related to greater self-efficacy [43]. Indeed, Phillips and Gully [42] found a serial mediation of self-efficacy and self-set goals between LoC and performance. Some researchers have already demonstrated the mediating role of self-efficacy between risk propensity [26, 28], emotional intelligence, proactive personality [26], Big-5 personality traits [44], entrepreneurial passion and creativity [31], on the one hand, and EI, on the other. Furthermore, Wilson and colleagues [45] have confirmed the key role played by self-efficacy in increasing both EI and actual entrepreneurial behaviour.

In the literature, there is no complete agreement as to whether a general or an entrepreneurial self-efficacy construct is more appropriate in relation to entrepreneurial outcomes [46]. Some authors have argued that general self-efficacy, the construct used in this study, may be sufficient, since it captures individuals’ perception of their ability to positively perform a variety of tasks across a variety of situations [47]. Indeed, entrepreneurship involves a broad range of roles, tasks, activities and competences [48], which may significantly vary across the different situations; therefore, a general self-efficacy measure is considered to apply more simply to entrepreneurship studies [48].

Supported by previous findings and Boyd and Vozikis’ model [7], the present study sought to investigate the mediational role of self-efficacy between personality traits, namely internal LoC and self-regulation, and EI.

Hypothesis 2: General self-efficacy is positively related with EI.

Hypothesis 3: General self-efficacy mediates the relation between internal LoC and self-regulation, on the one hand, and EI, on the other.

In order to offer a more comprehensive overview, this study also investigated the role of contextual factors. Regarding environmental factors, such as perceived barriers and support, numerous studies have suggested that they have an impact on EI [16, 20]. Some scholars have investigated the role of social networks [49] and prior family business exposure [27, 50], and some have analysed the impact of easy access to capital [16, 51, 52] and the availability of business information [16, 49]. Scholars have also focused on the role played by the educational system [16, 22, 53–55].

In our study, attention has been focused on the role played by family and friends in terms of affective, not material, support. Perceived support from family and friends refers to how much individuals consider themselves to be supported, sustained, and encouraged by relatives and friends when trying to become entrepreneurs [27]. Pruett and colleagues [56] conducted a study on a sample of American, Spanish and Chinese university students by considering social, cultural and psychological factors as predictors of EI. They found a strong effect of psychological factors, while also highlighting a significant relationship between family support and EI. This type of support (or lack of it) could be relevant: most people recognized family ties as close bonds [56]. Family could help when failures and/or mistakes occur, which may characterize the first phases of the entrepreneurial process [56]. Although support from intimate persons could play an important role, research on this topic is still lacking in literature. Therefore, we formulated the following hypotheses.

Hypothesis 4: Support from family and friends has a positive relation with EI.

Hypothesis 5: General self-efficacy mediates the relation between support from family and friends and EI.

In summary, the study intended to contribute to the literature on entrepreneurship and, specifically, EI in three ways. First of all, the study sought to confirm certain personality traits as determinants of EI, highlighting in particular the role of self-regulation, which has been less investigated to date [10, 29]. Second, sustained by Boyd and Vozikis’ model [7], the study intended to provide empirical support for the mediational role of self-efficacy between both personal and contextual factors and EI, and thus its key role in the entrepreneurial process. Third, what is primarily unique about the study is its investigation into support from family and friends, as one of the determinants of EI, thereby responding to the call for a more multidisciplinary and situational approach in research on entrepreneurship [6, 20].

### Gender differences

Globally, a gender gap in entrepreneurship and self-employment persists [57–59]. Despite the relevance of this issue, women’s lower propensity in entrepreneurial behaviour is not yet completely understood [57]. As Haus and colleagues [60] noted, some scholars have found higher levels of EI for males [28, 61]; however, other studies have not presented evidence to this effect, but instead investigated the role of gender stereotypes [62, 63]. In any case, it is widely recognized that the gender gap in entrepreneurship and self-employment exists at a global level and is explained by different contextual and situational factors [57] (gender role, discrimination in terms of market access [64], countries’ social norms in supporting entrepreneurship, human capital and education [45, 65, 66] and social capital [67–69]), as well as personal characteristics, such as self-efficacy [28, 45, 59, 66, 70], personality traits [57], risk-taking and fear of failure [59, 71, 72].

Koellinger and colleagues [59] found that the lower female propensity towards entrepreneurship is related to a lower level of confidence about their entrepreneurial capabilities, social network characteristics and a higher-level fear of failure. Atkinson and colleagues [58] suggested that one problem concerns the elusive nature of credibility, as revealed by female entrepreneurs, in terms of the need to be taken seriously. Wilson and colleagues [45] argued that entrepreneurial education increases the level of self-efficacy overall, but its impact is particularly strong in the case of women. Shinnar and colleagues [73] investigated gender differences in terms of the perceptions of barriers, concluding that women perceive lack of support (in China, the US and Belgium), fear of failure and lack of competency (in the US and Belgium) significantly more than men. Moreover, they found that gender moderated the relationship between the barrier related to perceived lack of support and the EI in different ways in the three countries investigated. Finally, Dabic and colleagues [74] found higher levels of perceived desirability and feasibility for men, compared with women, who are less self-confident, and more tense, reluctant and concerned about entrepreneurship, although they feel more supported by their families.

Since the literature refers to the presence of gender differences in entrepreneurship and EI, while reporting sometimes contradictory results, in this study, we took an explorative perspective and investigated potential differences between men and women at the level of EI and the other considered variables; moreover, we tested the hypothesized model across both groups.

## Methods

### The Italian context

To briefly describe the current state of entrepreneurship in Italy, some data elaborated by Global Entrepreneurship Monitor (GEM) in 2016 [75] have been used. Total Early-stage Entrepreneurial Activity (TEA) is an index developed by GEM, which measures the percentage of the adult population (18 to 64 years) who are in the process of starting or who have just started a business. According to the 2016 GEM survey [75], in Italy, the TEA index was equal to 4.4%; this rate was low, especially when compared with the same index for other European countries, such as the UK (8.8%), Romania (10.8%) or Portugal (8.2%) [75].

At the same time, in 2014, 11.4% of the Italian population declared an intention with regard to business creation [76]; this rate was higher in Italy in comparison with other countries, such as Germany (6.9%) and the UK (6.9%) [76], which could suggest that, while Italians are significantly inclined towards creating a business, in most cases, EI does not become an entrepreneurial choice and/or an entrepreneurial activity.

As for differences between men and women, the percentage of female entrepreneurs has increased in recent years within Italy; nevertheless, it remains below that for males [60, 70, 77, 78]. ISTAT data [79] confirmed that, in 2014, about 316,000 individuals launched a new business, of which 31.1% were female. According to the 2016 TEA index [75], the percentage of the female population in Italy, aged between 18 and 64 years, who were either a nascent entrepreneur or an owner-manager of a new business, was equal to 0.59%. Therefore, it is crucial to improve the understanding of which factors can support EI, particularly for Italian women.

### Participants and data collection procedure

A total of 658 participants from different Italian regions took part to this study; in particular, 319 (49%) were male and 339 were female (51%). The male sample aged between 20 and 68 years (*M* = 28.78; *SD* = 7.88). Among males, 35% were students, 23% were employees, 16% were self-employed workers, 19% were unemployed, the remaining had other occupations o were missing data. Regarding education, the most of male participants had a bachelor’s or master’s degree (58%) and 28% had a high school diploma.

The female sample aged between 20 and 55 years (*M* = 28.50; *SD* = 7.72). Among females, 38% were students, 28% were unemployed, 19% were employees, 9% were self-employed workers, the remaining had other occupations o were missing data. Regarding education, also the most of female participants had a bachelor’s or master’s degree (60%) and 28% had a high school diploma.

Participants completed the online self-report questionnaire STEPS (STartuppers and Entrepreneurs Potential Survey) promoted by an Italian no profit organization (Human Plus Foundation). The voluntary and not paid participation to the research, and the confidentiality of the data, were emphasized. We obtained informed consent by each participant. The study was conducted according to the Helsinki Declaration [80], and data protection followed regulation of the Italian country (Legislative Decree N. 196/2003).

### Measures

All items in the questionnaire were measured on a 7-point Likert-type scale (1 = very strongly disagree, 7 = very strongly agree). Items are available in S1 and S2 Questionnaires.

*Entrepreneurial intention (EI)* was measured by 5 items adapted from Liñán and Chen work [81]; an example item is “My professional goal is to become an entrepreneur”. Construct reliability (CR) in the whole sample was .98 and average variance extracted (AVE) in the whole sample was .73. Cronbach Alpha was .93 for the whole sample, .90 for the male sample and .94 for the female sample.

*General self-efficacy* was measured through 10 items [82]; an example item is “I am confident that I will succeed”. CR was .95 and AVE was .86. Cronbach Alpha was .88 for the whole sample, .86 for the male sample and .88 for the female sample.

*Internal LoC* was detached using 6 items [83]; an example item is “There is a direct link between a person’s abilities and the position he/she holds”. CR was .96 and AVE was .50. Cronbach Alpha was .79 for the whole sample, and .78 for both the female and male subgroups.

*Self-regulation* was measured by 8 items adapted from the work of Grasmick, Charles, Bursik, and Arneklev [84] and Tangney, Baumeister, & Boone [85]; an example item is “I often act without thinking through all the alternatives” (reverse item). CR was .88 and AVE was .50. Cronbach Alpha was .73 for the whole sample, .72 for the male sample and .75 for the female sample.

*Support from family and friends* was measured by 3 ad hoc items; an example is “People who are important for me think I should choose an entrepreneurial career”. CR was .98 and AVE was .78. Cronbach Alpha was .91 for the whole sample, .90 for the male sample and .92 for the female sample.

### Data analysis

The statistics software SPSS 24 was used to perform descriptive data analysis in each sample (male and female) separately. Pearson correlations were tested in order to examine the relationships among variables, and Cronbach’s alpha coefficient was calculated to test the reliability of each scale. The analysis of variance (*t*-test for independent samples) was used to examine differences in the variables’ means between male and female samples.

Since most of the variables considered in this study were personality-related, self-reports questionnaire was an appropriate method to detect them [86]. In order to address the common method variance and response bias issues we randomly inserted items into the questionnaire and we used a scale to assess social desirability, excluding all cases with low or high scores, according to the measure’s cut-off criteria [87]. Then, we conducted Harman’s single-factor test [88, 89] through a confirmatory factor analysis (CFA; ML solution). CFA results indicated that one single factor could not account for the variance in the data [χ^2^(464, N = 658) = 4398.02, *p* < .001, RMSEA = .11, CFI = .60, TLI = .57, SRMR = .11]. This indicated that common method variance was not a major problem in the present study.

In light of the high correlation we found between EI and support from family and friends in both groups [M: *r* = .60, *p* < .01; F: *r* = .78, *p* < .01], we tested discriminant validity between the two constructs following Anderson and Gerbing’s suggestions [90]. Thus, we constrained the estimated correlation parameter between the two variables to 1.0; then, we performed a chi-square difference test between the constrained and unconstrained models, founding a significantly lower χ^2^ value for the model in which the correlations were not constrained [Δχ^2^ = 638.43, *p* < .001]. This result suggested that the two variables were not perfectly correlated and that discriminant validity was achieved [90]. Moreover, to test the measurement model we performed a CFA which showed a good fit to the data [χ^2^(454, N = 658) = 1171.52, *p* < .001, RMSEA = .05 (.05, .05), CFI = .93, TLI = .92, SRMR = .06] and we tested AVE and CR for each construct, as previously reported for each measure.

A multi-group full structural equation model (SEM) was performed using Mplus 7 in order to test the hypothesized model. Age was used as control variable; since it presented some significant correlations in the female group, it was considered in the SEM as independent variable. For reasons of parsimony, item parcelling technique [91], which allows parcel creation starting from different items referring to a same construct, has been applied for self-regulation and self-efficacy variables. The method of estimation was Maximum Likelihood (ML). According to the literature [92], the model was assessed by several goodness-of-fit criteria: the χ^2^ goodness-of-fit statistic; the Root Mean Square Error of Approximation (RMSEA); the Comparative Fit Index (CFI); the Tucker Lewis Index (TLI); the Standardized Root Mean Square Residual (SRMR); the Akaike’s Information Criterion (AIC). Finally, bootstrapping procedure was used to test the significance of the mediation effects [93].

## Results

### Descriptive analysis, analysis of variance and correlations

Analysis of variance between male and female samples showed significant differences for all considered variables, except for self-regulation: males showed higher level of EI [*t* (636) = 9.15, *p* < .001], general self-efficacy [*t* (645) = 6.11, *p* < .001], internal LoC [*t* (656) = 4.42, *p* < .001] and support from family and friends [*t* (655) = 4.96, *p* < .001]. As regards self-regulation, the difference between males and females was not significant [*t* (656) = 1.94, *p* = .053]. Table 1 shows means, standard deviations and *t*-test results.

**Table 1:** Means, standard deviations, *t*-test results for males (n = 319) and females (n = 339).

	Male		Female		*t*-test
	*M*	*SD*	*M*	*SD*	
**1. Entrepreneurial intention**	5.16	1.42	4.00	1.81	*t* (636) = 9.15, *p* < .001
**2. General self-efficacy**	5.56	.74	5.17	.90	*t* (645) = 6.11, *p* < .001
**3. Internal locus of control**	4.95	1.05	4.58	1.07	*t* (656) = 4.42, *p* < .001
**4. Self-regulation**	4.36	.92	4.22	.99	*t* (656) = 1.94, *p* = .053
**5. Support from family and friends**	4.23	1.70	3.54	1.89	*t* (655) = 4.96, *p* < .001

Table 2 shows correlations among the study variables and internal consistency of each scale in the whole sample and Table 3 shows the same statistics separately for male and female groups. All *α* values meet the criterion of .70 [94] as they ranged between .72 and .94. All the main significant correlations between the variables were in line with the expected directions in both groups.

**Table 2:** Cronbach’s alphas and correlations among the study variables in the whole sample (n = 658).

	*1*	*2*	*3*	*4*	*5*	*6*
**1. Entrepreneurial intention**	.*93*					
**2. General self-efficacy**	.57**	.*88*				
**3. Internal locus of control**	.30**	.40**	.*79*			
**4. Self-regulation**	.12**	.15**	-.04	.*73*		
**5. Support from family and friends**	.73**	.52**	.26**	.07	.*91*	
**6. Age**	-.01	-.01	-.10**	.12**	.03	–

Notes. Cronbach’s *α* for the whole sample on the diagonal.

** *p* < .01.

**Table 3:** Cronbach’s alphas and correlations among the study variables for males (n = 319) and females (n = 339).

	*1*	*2*	*3*	*4*	*5*	*6*
**1. Entrepreneurial intention**	.*90/*.*94*	.54**	.25**	.05	.78**	.02
**2. General self-efficacy**	.54**	.*86/*.*88*	.40**	.07	.54**	.02
**3. Internal locus of control**	.29**	.34**	.*78/*.*78*	-.19**	.22**	-.14*
**4. Self-regulation**	.17**	.23**	.10	.*72/*.*75*	.03	.21*
**5. Support from family and friends**	.60**	.44**	.24**	.10	.*90/*.*92*	.01
**6. Age**	-.05	-.05	-.08	.03	.04	–

Notes. Correlations for the male group below the diagonal; correlations for the female group above the diagonal. Cronbach’s *α* for male/female sample on the diagonal.

* *p* < .05.

** *p* < .01.

### Multi-group structural equation model

The multi-group full SEM of the hypothesized model with all parameters constrained to be equal across groups, namely M_1_, fitted to the data well: *χ*^2^ (319, N_Male_ = 319, N_Female_ = 339) = 638.05, *p* < .001, CFI = .95, TLI = .95, RMSEA = .06 (90% CI .05, .06), SRMR = .07. As Table 4 shows, the model without mediation (M_2_) which investigated the relation between general self-efficacy, self-regulation, internal LoC, support from family and friends and age, on the one hand, and EI, on the other one, had not a significantly better fit to the data than M_1_. Moreover, a model with self-regulation instead of self-efficacy as mediator (M_3_) showed a significantly worst fit to the data compared with M_1_. Therefore, the hypothesized model with general self-efficacy as mediator was the best one.

**Table 4:** Results of alternative multi-group SEMs.

	χ^2^	*df*	*p*	CFI	TLI	RMSEA	SRMR	AIC	Comparison	Δ*χ*^*2*^	*p*
**M**_**1**_.	638.05	319	< .01	.95	.95	.06 (.05, .06)	.07	41847.01			
**M**_**2**_.	634.31	317	< .01	.95	.95	.06 (.05, .06)	.07	41847.27	M_1_–M_2_	3.74	> .05
**M**_**3**_.	654.32	319	< .01	.95	.95	.06 (.05, .06)	.09	41863.28	M_3_–M_1_	16.27	< .01
**M**_**4**_.	600.39	316	< .01	.96	.95	.05 (.05, .06)	.06	41815.35	M_1_–M_4_	37.66	< .01

Note.

M_1_. Hypothesized constrained model with general self-efficacy as mediator.

M_2_. No mediation model.

M_3_. Constrained model with self-regulation as mediator.

M_4_. Constrained model with general self-efficacy as mediator and 3 released parameters (see Table 5).

**Fig 1:**
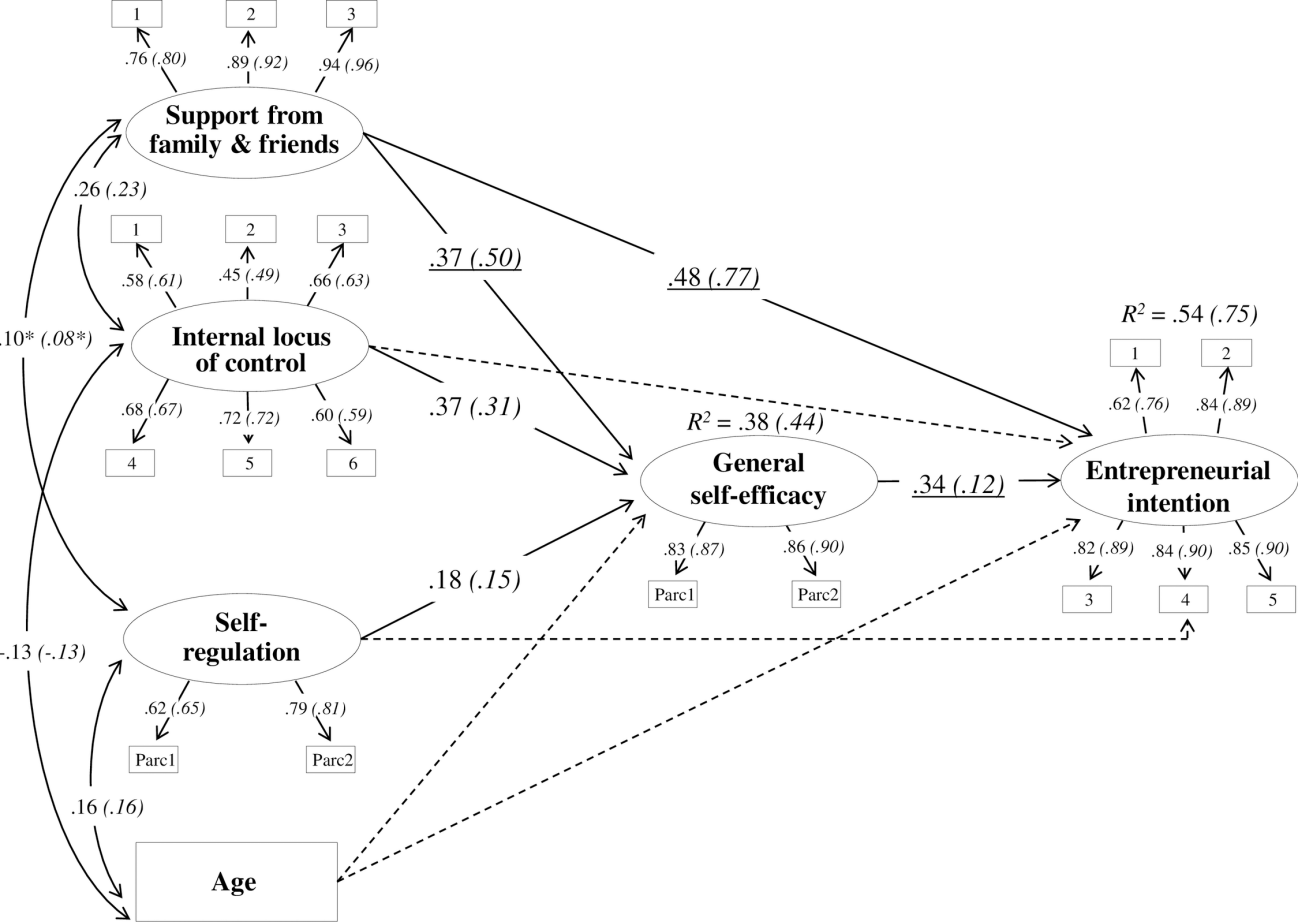
The final model (standardized path coefficients, *p* < .001; *p < .01). Male sample data are out of parentheses, female sample data are in parentheses. Underlined data are statistically different between men and women. Discontinuous lines indicate non-significant relationships.

Examination of the modification indices of M_1_ revealed that sequentially releasing the equality constraints of specific structural parameters and retesting the model it resulted in a better overall model. Table 5 shows the constraints released and the decrement in fit indices (χ^2^ and AIC). The final model (M_4_) with 3 free structural parameters had the best fit to the data and is shown in Fig 1. In the final model, the latent variables were well defined with factor loadings of the observed variables being greater than .45. The model presented a significant positive relationship between both internal LoC [M: β = .37, *p* < .001; F: β = .31, *p* < .001] and self-regulation [M: β = .18, *p* < .001; F: β = .15, *p* < .001] and general self-efficacy, in both samples. Internal LoC and self-regulation did not present a significant relationship with EI. Support from family and friends showed a positive relationship with both general self-efficacy [M: β = .37, *p* < .001; F: β = .50, *p* < .001] and EI [M: β = .48, *p* < .001; F: β = .77, *p* < .001], significantly stronger for women in both cases. The relationship between general self-efficacy and EI was significant and positive [M: β = .34, *p* < .001; F: β = .12, *p* < .001], significantly stronger for men. Support from family and friends positively correlated with both internal LoC [M: r = .26, *p* < .001; F: r = .23, *p* < .001] and self-regulation [M: r = .10, *p* < .01; F: r = .08, *p* < .01]. Finally, age correlated positively with self-regulation [M: r = .16, *p* < .001; F: r = .16, *p* < .001] and negatively with internal LoC [M: r = -.13, *p* < .001; F: r = -.13, *p* < .001]; age did not show any relationship with the two endogenous variables. The model explained about 54% and 75% of the variation in EI respectively for men and women, and 38% and 44% of the variation in general self-efficacy respectively for men and women.

**Table 5:** Structural parameters sequentially released and fit indices of nested models.

Released parameters	χ^2^	*df*	*p*	CFI	TLI	RMSEA	SRMR	AIC	Δ*χ*^*2*^	*p*
M_1_. All parameters constrained	638.05	319	< .01	.95	.95	.06 (.05, .06)	.07	41847.01		
FF Supp → EI	612.49	318	< .01	.96	.95	.05 (.05, .06)	.07	41823.45	25.56	< .01
G-Self-Eff → EI	605.08	317	< .01	.96	.95	.05 (.05, .06)	.06	41818.04	7.41	< .01
M_4_. FF Supp → G-Self-Eff	600.39	316	< .01	.96	.95	.05 (.05, .06)	.06	41815.35	4.69	< .05

Note.

M_1_. Hypothesized constrained model with self-efficacy as mediator.

FF Supp: Support from family and friends. EI: Entrepreneurial intention. Self-Reg: Self-regulation. Int LoC: Internal locus of control. G-Self-Eff: General self-efficacy.

The mediating paths and indirect effects were evaluated using a bootstrapping procedure that extracted 10,000 new samples from the original one and calculated all direct and indirect parameters of the model [95]. A significant mediation occurs when the confidence interval does not include zero. Results in Table 6 show that all the mediated effects were statistically significant for both samples and were higher for males than for females. According to the bootstrapping procedure, general self-efficacy fully mediated the relationship between internal LoC and self-regulation, on the one hand, and EI, on the other. Moreover, general self-efficacy partially mediated the relationship between support from family and friends and EI.

**Table 6:** Indirect effects using bootstrapping (10,000 replications).

**Indirect effects—male sample**	Bootstrap			
	Est.	S.E.	*p*	CI 95%
Int Loc → G-Self-Eff → EI	.12	.03	.000	(.06, .18)
Self-Reg → G-Self-Eff → EI	.06	.02	.012	(.01, .11)
FF Supp → G-Self-Eff → EI	.13	.03	.000	(.06, .19)
**Indirect effects—female sample**	Bootstrap			
	Est.	S.E.	*p*	CI 95%
Int Loc → G-Self-Eff → EI	.04	.02	.020	(.01, .07)
Self-Reg → G-Self-Eff → EI	.02	.01	.049	(.01, .04)
FF Supp → G-Self-Eff → EI	.06	.02	.015	(.01, .11)

*Note*. All parameter estimates are presented as standardized coefficients. CI = confidence interval. Int LoC: Internal locus of control. G-Self-Eff: General self-efficacy. EI: Entrepreneurial intention. Self-Reg: Self-regulation. FF Supp: Support from family and friends.

## Discussion

This study aimed to investigate the determinants of EI by considering both personal and contextual factors, according to the LFM [20], as well as adopting a multidisciplinary and situational perspective [6, 8]. Studying the determinants of EI in an Italian sample, the research focused on the key mediational role of general self-efficacy, according to Boyd and Vozikis’ model [7], and explored gender differences.

Among the personal characteristics, we only found a direct relationship with EI in the case of general self-efficacy, confirming Hypothesis 2, but not Hypothesis 1. The relation between self-efficacy and EI was significantly stronger for men than women, indicating that different variables explain EI development in the two categories. As for internal LoC and self-regulation, the relation with EI was totally mediated by self-efficacy, in line with Boyd and Vozikis’ model [7]. Therefore, Hypothesis 3 was confirmed for both men and women, thereby contributing new knowledge to the literature about determinants of EI, in particular, by highlighting the role of self-regulation, a personality trait that few researchers have investigated to date.

Hypotheses 4 and 5 were informed by the need to investigate the role of a specific contextual factor, support from family and friends, in relation to EI, another dynamic underexplored in previous studies. Both hypotheses were confirmed, since we found both a direct relationship and one mediated by general self-efficacy between support from family and friends and EI in male and female samples. Further, despite the level of support from family and friends being significantly higher for men, the relation between this form of support and both self-efficacy and EI was stronger for women. This can be considered as a significant and original finding of this study, which indicates that, for women, perceiving external support from important people is crucial in the development of EI, more than having high levels of personal characteristics, such as self-efficacy. A previous study, which considered material support from family, found that, for women, having access to socio-economic family resources seems to have a direct impact on whether or they start their own business [96]. Perhaps gender bias within financial institutions makes it harder for women to obtain the financial resources needed to start their own business [97, 98], in turn making parental support a crucial element. The present study also highlighted that perceived affective support may have an incentive function, which may contrast with those typical gender stereotypes related to entrepreneurship that could hinder an entrepreneurial career for women. Indeed, the common idea is that entrepreneurship, like business in general, involves male-gendered concepts with masculine connotations [99].

In summary, as for gender, many differences have been found. First of all, the study confirmed higher levels of EI for men compared with women [28, 60, 61]; moreover, men showed higher levels of self-efficacy, internal LoC and support from family and friends. Furthermore, gender showed a buffering role in the relationship between such support and both self-efficacy and EI. To the best of our knowledge, this is the first study that has highlighted the positive role that support from family and friends may play, particularly for women, in increasing the two constructs. Moreover, we found that the relationship between self-efficacy and EI was stronger for men, a finding that could extend knowledge in the relevant literature, in which contradictory results on this aspect are found [28, 45, 59, 66, 70]. All in all, these results seem to confirm that the old social roles associated with men and women still exist in Italy, including in relation to entrepreneurial choice.

Finally, the study used age as a control variable in the SEM, although no significant relationships with the two endogenous variables were found. Nevertheless, age showed a positive correlation with self-regulation and a negative one with internal LoC, a result, the latter, that could be regarded as noteworthy. The perception of control across the lifespan may change, possibly affected by situational factors and personal characteristics, as suggested by Fitch and Slivinske [100]: incongruence between needs and supplies, as well as demands and resources, could gradually increase the perception of a lack of control over time. This aspect could be reinforced by the working context, particularly if it is perceived to be unfair and incongruent in terms of career opportunities in due course, as often happens for Italian women.

### Limitations and future research

As for the study limitations, the main one is related to its cross-sectional design, which did not allow us to establish causal relations between variables [101]. Considering the nature of the hypotheses of this study, and particularly the debated potential overlap between LoC and self-efficacy [102], future research should apply longitudinal approaches in order to further examine the effects among variables and their influence on EI over time. Longitudinal studies should also consider subsequent phases of the entrepreneurial process by investigating the relationship between EI, entrepreneurial actions and success. In this way, the buffering role of self-efficacy between intention and entrepreneurial actions, supported by Boyd and Vozikis [7], could be investigated, alongside the relationship between intention and behaviour, according to the TPB [18, 103] and Bird’s IEI model [13].

A second limitation concerns the use of self-reported data, which could have potentially inflated results [104], given respondents’ tendency to answer in a consistent manner. In future studies, it would be interesting to also consider other-reported (especially involving family and friends).

Furthermore, the convenience sampling procedure could be considered a limitation, since the study’s findings are not generalizable to the Italian population. Although it is difficult to cover the entire country, future studies should try to involve participants from all the Italian regions, as well as exploring differences between the north and south of Italy. Indeed, resources for entrepreneurs are more available in the north, compared with the south, where gender stereotypes are also still stronger [105].

Finally, the present study inevitably overlooked certain EI determinants. Thus, future studies could consider the role of other contextual factors, such as access to capital [16, 51, 52] and bureaucratic barriers [106], which are critical aspects for Italian entrepreneurs. As the educational system may also play an important role [16, 22, 53–55], its study would be of particular interest in Italy, where very little attention is dedicated to entrepreneurship at the school and university level. Moreover, this study employed a general self-efficacy construct; as previously mentioned, some authors have suggested its application [48], while others have considered entrepreneurial self-efficacy measures to be more appropriate in the entrepreneurship context (e.g., [28]), on the basis that the more task-specific the measurement of self-efficacy is, the better its predictive role in research on the task-specific outcomes of interest [107]. Future studies could further investigate the mediation role of self-efficacy, as highlighted by this research, by considering an entrepreneurial self-efficacy measure.

### Conclusions and practical implications

In conclusion, this study contributes to entrepreneurship research in three ways. First, it showed that internal LoC, self-regulation and self-efficacy are determinants of EI, thereby confirming the key role of self-efficacy as a motivational characteristic capable of mediating the relation between personal and context factors and EI. These results have important implications for entrepreneurship professionals and educators. Specific and effective training and education, particularly for students, could be provided in order to enhance their beliefs about their capacity to become entrepreneurs. These beliefs should be strengthened through an internal tendency to explain events (internal LoC [32]) and a sense of control over one’s own thoughts, motivations, and behaviours towards goal achievements (self-regulation [35]). Beside the more traditional methods of teaching entrepreneurship and theories [28], training and education programmes should consist of practical learning opportunities, such as business plan writing and case studies [66]. Moreover, training and coaching sessions could support the development of self-awareness and the improvements in the above-mentioned personal characteristics [29].

Second, the study underlined the important role of perceived support from family and friends as a determinant of EI for aspiring Italian entrepreneurs, confirming that a focus on personal characteristics alone is not sufficient for gaining a better understanding of EI and career [6]. This result suggests that families, and people in general, could be a resource capable of fostering and supporting EI. In light of this, there is a need to develop a social culture oriented towards entrepreneurship, especially in Italy. To this extent, the mainstream media should increase the level of knowledge and awareness about entrepreneurial careers among its consumers.

The findings further suggested a gendered process view of EI, as men and women appear to draw on different sources of support: this represents the third contribution made by this study. These differences could be a consequence of gender stereotypes associated with the entrepreneur role [99]. In this context, the mainstream media, as well as educators, could have an impact by providing varied information that associates entrepreneurship with gender-neutral characteristics, i.e., attributable to both men and women [99]. Moreover, in order to support female entrepreneurs, training and education should be customized to meet both genders’ needs [74].

## Supporting information

S1 Questionnaire English version. English version of the questionnaire used in the study.
(DOCX)

S2 Questionnaire Italian version. Italian version of the questionnaire used in the study.
(DOCX)

S1 Dataset SPSS dataset. Data used in the study.
(SAV)
