# Supplementary material for: Personality and social support as determinants of entrepreneurial intention. Gender differences in Italy
Source: PLoS One. 2018 Jun 28;13(6):e0199924. doi: 10.1371/journal.pone.0199924 (PMC6023202; doi:10.1371/journal.pone.0199924)
Supplement: S1 Questionnaire — English version of the questionnaire used in the study. (DOCX) [file pone.0199924.s001.docx]

**QUESTIONNAIRE – English version**

**1. Age: _**___________

**2. Gender:**

1. ☐ Male
2. ☐ Female

**3. What is the higher level of education that you have achieved?**

1. ☐ Primary school diploma
2. ☐ Certificate for secondary education – middle school
3. ☐ High school diploma
4. ☐ Bachelor’s degree
5. ☐ Unique cycle degree or Master degree
6. ☐ II level Master degree
7. ☐ PhD
8. ☐ Other (specify)_______________________________________________________________

**4. At present, your main employment is:**

1. ☐ Full-time employment
2. ☐ Part-time employment
3. ☐ Independent contractor/self-employment
4. ☐ Jobseekers
5. ☐ Unemployment
6. ☐ Retiree
7. ☐ Students
8. ☐ Other (specify)_______________________________________________________________

**5. Indicate on a scale from 1 to 7 how much you agree with each of the following statements.**

(1 = very strongly disagree, 7 = very strongly agree)

|  | **1** | **2** | **3** | **4** | **5** | **6** | **7** |
| --- | --- | --- | --- | --- | --- | --- | --- |
| 1. I am ready to do anything to be an entrepreneur. | ➀ | ➁ | ➂ | ➃ | ➄ | ➅ | ➆ |
| 1. My professional goal is to become an entrepreneur. | ➀ | ➁ | ➂ | ➃ | ➄ | ➅ | ➆ |
| 1. I am determined to create a firm in the future. | ➀ | ➁ | ➂ | ➃ | ➄ | ➅ | ➆ |
| 1. I have very seriously thought of starting a firm. | ➀ | ➁ | ➂ | ➃ | ➄ | ➅ | ➆ |
| 1. I have the strong intention to start a firm someday. | ➀ | ➁ | ➂ | ➃ | ➄ | ➅ | ➆ |
| 1. My family members have always thought I should choose an entrepreneurial career. | ➀ | ➁ | ➂ | ➃ | ➄ | ➅ | ➆ |
| 1. My friends think that I should choose an entrepreneurial career. | ➀ | ➁ | ➂ | ➃ | ➄ | ➅ | ➆ |
| 1. People who are important for me think that I should choose an entrepreneurial career. | ➀ | ➁ | ➂ | ➃ | ➄ | ➅ | ➆ |

**6. Thinking about you, indicate how much you agree with each of the following statements.**

(1 = very strongly disagree, 7 = very strongly agree)

| 1 | I think I will always be able to reach my objective even if I must carry out a difficult task. | ➀ ➁ ➂ ➃ ➄ ➅ ➆ |
| --- | --- | --- |
| 2 | I always prove to be very smart | ➀ ➁ ➂ ➃ ➄ ➅ ➆ |
| 3 | It always ends with earning in proportion to your value/worth | ➀ ➁ ➂ ➃ ➄ ➅ ➆ |
| 4 | When faced with new tasks and challenges I have always been confident of being able to complete them | ➀ ➁ ➂ ➃ ➄ ➅ ➆ |
| 5 | I am a mature and even-tempered person | ➀ ➁ ➂ ➃ ➄ ➅ ➆ |
| 6 | I am confident that I will succeed | ➀ ➁ ➂ ➃ ➄ ➅ ➆ |
| 7 | I change my mind quite often | ➀ ➁ ➂ ➃ ➄ ➅ ➆ |
| 8 | I have always behaved honestly and loyally with others | ➀ ➁ ➂ ➃ ➄ ➅ ➆ |
| 9 | When I pursue something I almost always obtain better results than others | ➀ ➁ ➂ ➃ ➄ ➅ ➆ |
| 10 | Others would describe me as an impulsive person | ➀ ➁ ➂ ➃ ➄ ➅ ➆ |
| 11 | I am immediately well-liked by everyone | ➀ ➁ ➂ ➃ ➄ ➅ ➆ |
| 12 | Earnings are above all the result of hard work | ➀ ➁ ➂ ➃ ➄ ➅ ➆ |
| 13 | When I take a test or an exam I am convinced I can overcome it positively | ➀ ➁ ➂ ➃ ➄ ➅ ➆ |
| 14 | I would like to have more self-discipline | ➀ ➁ ➂ ➃ ➄ ➅ ➆ |
| 15 | In the various fields in which I have worked I have always demonstrated great competence and capacity |  |
| 16 | There is a direct link between a person’s abilities and the position he/she holds | ➀ ➁ ➂ ➃ ➄ ➅ ➆ |
| 17 | I am confident that my results will be appreciated and recognized by others | ➀ ➁ ➂ ➃ ➄ ➅ ➆ |
| 18 | I get carried away by my feelings | ➀ ➁ ➂ ➃ ➄ ➅ ➆ |
| 19 | I tackle everything with extraordinary bravery | ➀ ➁ ➂ ➃ ➄ ➅ ➆ |
| 20 | I am always confident when I am faced difficult tasks | ➀ ➁ ➂ ➃ ➄ ➅ ➆ |
| 21 | I always prove great value in every activity that I do | ➀ ➁ ➂ ➃ ➄ ➅ ➆ |
| 22 | Generally, people who work well obtain rewards | ➀ ➁ ➂ ➃ ➄ ➅ ➆ |
| 23 | I do not feel troubled when faced with any situation, because until now I have always been able to work it out with my capabilities | ➀ ➁ ➂ ➃ ➄ ➅ ➆ |
| 24 | Sometimes I am unable to avoid doing something, even if I know it is wrong | ➀ ➁ ➂ ➃ ➄ ➅ ➆ |
| 25 | I always provide exceptional or out of the ordinary performances | ➀ ➁ ➂ ➃ ➄ ➅ ➆ |
| 26 | Promotions are allocated to people who work well | ➀ ➁ ➂ ➃ ➄ ➅ ➆ |
| 27 | I have never had trouble immediately understanding and coping with even the most complicated situations | ➀ ➁ ➂ ➃ ➄ ➅ ➆ |
| 28 | I often act without thinking through all the alternatives | ➀ ➁ ➂ ➃ ➄ ➅ ➆ |
| 29 | I never betray the trust granted to me by others | ➀ ➁ ➂ ➃ ➄ ➅ ➆ |
| 30 | I think I comprehend the core of the matter before others | ➀ ➁ ➂ ➃ ➄ ➅ ➆ |
| 31 | I often do things that seem to me right in the present, even if at the cost of future objectives | ➀ ➁ ➂ ➃ ➄ ➅ ➆ |
| 32 | I am an agreeable person with whom everyone is comfortable | ➀ ➁ ➂ ➃ ➄ ➅ ➆ |
| 33 | A well prepared person always finds a satisfying job | ➀ ➁ ➂ ➃ ➄ ➅ ➆ |
| 34 | When I pursue an objective I hardly change my route, even if I realize that it is not the best way | ➀ ➁ ➂ ➃ ➄ ➅ ➆ |

**The questionnaire is finished, thank you for your collaboration!**
