# Supplementary material for: Personality and social support as determinants of entrepreneurial intention. Gender differences in Italy
Source: PLoS One. 2018 Jun 28;13(6):e0199924. doi: 10.1371/journal.pone.0199924 (PMC6023202; doi:10.1371/journal.pone.0199924)
Supplement: S2 Questionnaire — Italian version of the questionnaire used in the study. (DOCX) [file pone.0199924.s002.docx]

**QUESTIONARIO – versione italiana**

**1. Età:** ____________

**2. Genere:**

1. ☐Maschio
2. ☐Femmina

**3. Qual è il più alto titolo di studio da te conseguito?**

1. ☐ Diploma di scuola primaria
2. ☐ Diploma di scuola secondaria di primo grado – medie
3. ☐ Diploma di scuola secondaria di secondo grado – superiori
4. ☐ Laurea breve/Laurea triennale
5. ☐ Laurea quinquennale ciclo unico/Laurea magistrale
6. ☐ Master post-laurea
7. ☐ Dottorato di ricerca
8. ☐ Altro (specificare)_______________________________________________________________

**4. Attualmente la tua occupazione principale è:**

1. ☐ Lavoratore dipendente full-time
2. ☐ Lavoratore dipendente part-time
3. ☐ Libero professionista/lavoratore autonomo
4. ☐ In cerca di prima occupazione
5. ☐ Disoccupato
6. ☐ Pensionato
7. ☐ Studente
8. ☐ Altro (specificare)_______________________________________________________________

**5. Indica su una scala da uno a sette quanto sei d’accordo con ciascuna delle seguenti affermazioni.**

(1 = totalmente in disaccordo; 7 = pienamente d’accordo)

|  | **1** | **2** | **3** | **4** | **5** | **6** | **7** |
| --- | --- | --- | --- | --- | --- | --- | --- |
| 1. Sono pronto a fare qualunque cosa pur di diventare un imprenditore | ➀ | ➁ | ➂ | ➃ | ➄ | ➅ | ➆ |
| 1. Il mio obiettivo professionale è di diventare un imprenditore | ➀ | ➁ | ➂ | ➃ | ➄ | ➅ | ➆ |
| 1. Sono determinato a creare una start up in futuro | ➀ | ➁ | ➂ | ➃ | ➄ | ➅ | ➆ |
| 1. Ho pensato seriamente di fondare una nuova impresa | ➀ | ➁ | ➂ | ➃ | ➄ | ➅ | ➆ |
| 1. Ho la ferma intenzione di avviare un'impresa prima o poi | ➀ | ➁ | ➂ | ➃ | ➄ | ➅ | ➆ |
| 1. I membri della mia famiglia hanno sempre pensato che dovrei intraprendere la carriera di imprenditore | ➀ | ➁ | ➂ | ➃ | ➄ | ➅ | ➆ |
| 1. I miei amici pensano che dovrei intraprendere la carriera di imprenditore | ➀ | ➁ | ➂ | ➃ | ➄ | ➅ | ➆ |
| 1. Le persone che sono importanti per me pensano che dovrei intraprendere la carriera di imprenditore | ➀ | ➁ | ➂ | ➃ | ➄ | ➅ | ➆ |

**6. Riflettendo su di te, indica il tuo grado di accordo rispetto alle seguenti affermazioni.**

(1 = per niente d’accordo; 7 = del tutto d’accordo)

| 1 | Penso che riuscirò sempre a raggiungere l'obiettivo anche se devo svolgere un compito difficile | ➀ ➁ ➂ ➃ ➄ ➅ ➆ |
| --- | --- | --- |
| 2 | Do sempre prova di grande intelligenza | ➀ ➁ ➂ ➃ ➄ ➅ ➆ |
| 3 | Si finisce sempre con il guadagnare in proporzione a quanto si vale | ➀ ➁ ➂ ➃ ➄ ➅ ➆ |
| 4 | Di fronte a compiti e sfide nuove sono sempre stato fiducioso di riuscire a portarli a termine | ➀ ➁ ➂ ➃ ➄ ➅ ➆ |
| 5 | Sono una persona molto matura ed equilibrata | ➀ ➁ ➂ ➃ ➄ ➅ ➆ |
| 6 | Sono convinto che avrò successo | ➀ ➁ ➂ ➃ ➄ ➅ ➆ |
| 7 | Cambio idea piuttosto spesso | ➀ ➁ ➂ ➃ ➄ ➅ ➆ |
| 8 | Mi sono sempre comportato/a in modo onesto e leale con gli altri | ➀ ➁ ➂ ➃ ➄ ➅ ➆ |
| 9 | Quando mi prefiggo qualcosa ottengo quasi sempre risultati migliori degli altri | ➀ ➁ ➂ ➃ ➄ ➅ ➆ |
| 10 | Gli altri mi descriverebbero come una persona impulsiva | ➀ ➁ ➂ ➃ ➄ ➅ ➆ |
| 11 | Risulto subito simpatico a tutti | ➀ ➁ ➂ ➃ ➄ ➅ ➆ |
| 12 | Il guadagno è frutto soprattutto di duro lavoro | ➀ ➁ ➂ ➃ ➄ ➅ ➆ |
| 13 | Quando sostengo una prova o un esame sono convinto di poterlo superare positivamente | ➀ ➁ ➂ ➃ ➄ ➅ ➆ |
| 14 | Vorrei avere una maggiore autodisciplina | ➀ ➁ ➂ ➃ ➄ ➅ ➆ |
| 15 | Nei diversi campi in cui ho operato ho dimostrato sempre grande competenza e capacità | ➀ ➁ ➂ ➃ ➄ ➅ ➆ |
| 16 | Esiste un rapporto diretto tra le capacità di una persona e il posto che ricopre | ➀ ➁ ➂ ➃ ➄ ➅ ➆ |
| 17 | Ho fiducia nel fatto che i miei risultati saranno riconosciuti e apprezzati dagli altri | ➀ ➁ ➂ ➃ ➄ ➅ ➆ |
| 18 | Mi lascio trasportare dai miei sentimenti | ➀ ➁ ➂ ➃ ➄ ➅ ➆ |
| 19 | Affronto ogni cosa con estremo coraggio | ➀ ➁ ➂ ➃ ➄ ➅ ➆ |
| 20 | Sono sempre fiducioso davanti a compiti difficili | ➀ ➁ ➂ ➃ ➄ ➅ ➆ |
| 21 | Dimostro grande valore in ogni attività che intraprendo | ➀ ➁ ➂ ➃ ➄ ➅ ➆ |
| 22 | Generalmente le persone che lavorano bene ottengono delle ricompense | ➀ ➁ ➂ ➃ ➄ ➅ ➆ |
| 23 | Non mi sento in difficoltà davanti a nessuna situazione, poiché finora sono sempre riuscito a cavarmela con le mie capacità | ➀ ➁ ➂ ➃ ➄ ➅ ➆ |
| 24 | A volte non riesco a evitare di fare qualcosa, anche se so che è sbagliato | ➀ ➁ ➂ ➃ ➄ ➅ ➆ |
| 25 | Fornisco sempre prestazioni eccezionali o fuori dal comune | ➀ ➁ ➂ ➃ ➄ ➅ ➆ |
| 26 | Le promozioni vengono assegnate alle persone che lavorano bene | ➀ ➁ ➂ ➃ ➄ ➅ ➆ |
| 27 | Non ho mai avuto problemi a capire immediatamente e fronteggiare anche le situazioni più complicate | ➀ ➁ ➂ ➃ ➄ ➅ ➆ |
| 28 | Spesso agisco senza pensare a tutte le alternative | ➀ ➁ ➂ ➃ ➄ ➅ ➆ |
| 29 | Non tradisco mai la fiducia accordatami dagli altri | ➀ ➁ ➂ ➃ ➄ ➅ ➆ |
| 30 | Credo di afferrare prima degli altri il nocciolo della questione | ➀ ➁ ➂ ➃ ➄ ➅ ➆ |
| 31 | Spesso faccio cose che mi sembrano giuste nel presente, anche a spese di obiettivi futuri | ➀ ➁ ➂ ➃ ➄ ➅ ➆ |
| 32 | Sono una persona piacevole con la quale tutti stanno a proprio agio | ➀ ➁ ➂ ➃ ➄ ➅ ➆ |
| 33 | Una persona ben preparata trova sempre un lavoro soddisfacente | ➀ ➁ ➂ ➃ ➄ ➅ ➆ |
| 34 | Quando perseguo un obiettivo difficilmente cambio strada, anche se mi rendo conto che non è la strada migliore | ➀ ➁ ➂ ➃ ➄ ➅ ➆ |

**Il questionario è finito, grazie per la collaborazione!**
